# Supplementary material for: Electrode interface optimization advances conversion efficiency and stability of thermoelectric devices
Source: Nat Commun. 2020 Jun 1;11:2723. doi: 10.1038/s41467-020-16508-x (PMC7264234; doi:10.1038/s41467-020-16508-x)
Supplement: Supplementary file 1 — Supplementary Information [file 41467_2020_16508_MOESM1_ESM.pdf]

Supplementary Information

Chu, et al. Electrode interface optimization advances conversion efficiency and stability of thermoelectric devices. Nat. Comms. (2020).

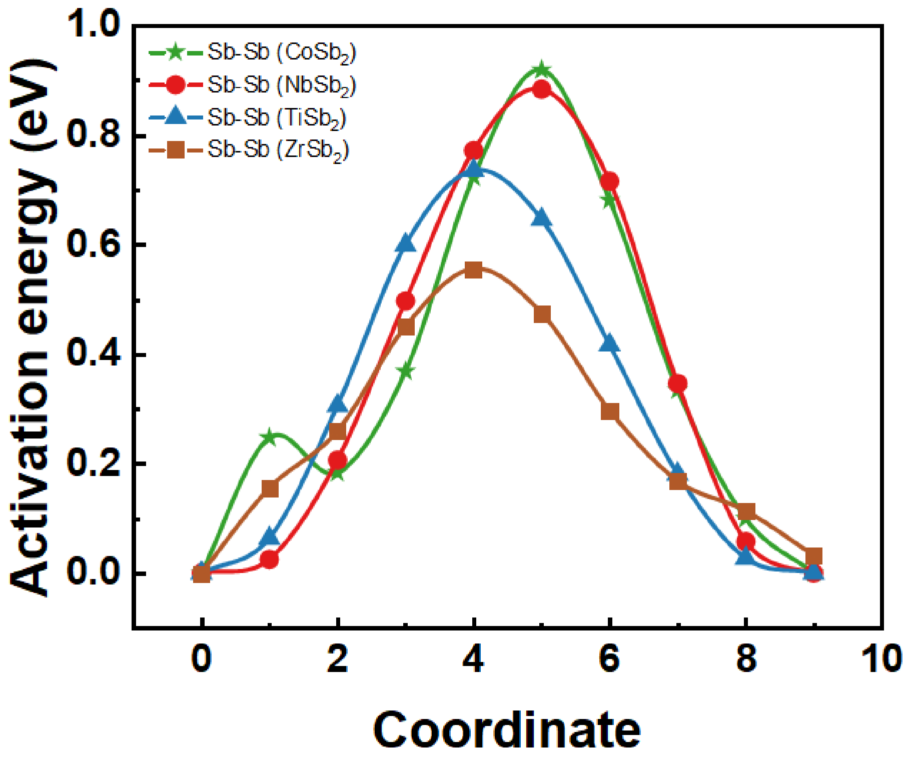

Supplementary Figure 1 The activation energy in XSb_y compounds for various diffusion channels: Sb atom to Sb vacancy site (Sb-Sb) in $CoSb_2$ and XSb_y compounds. Here X refers *d*-metal atom.

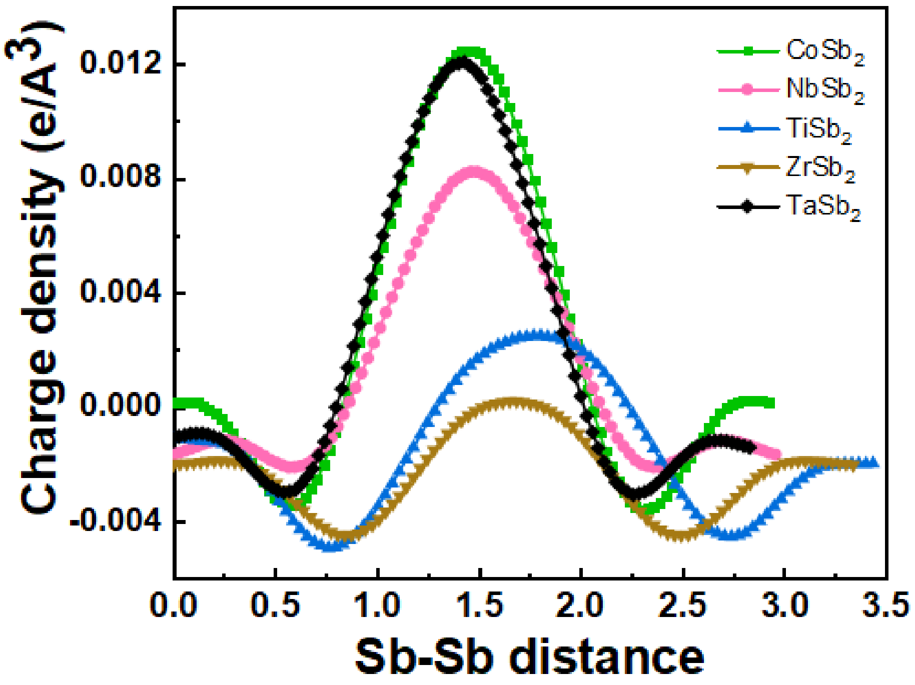

Supplementary Figure 2 Line profiles of charge density from one Sb site to the other Sb.

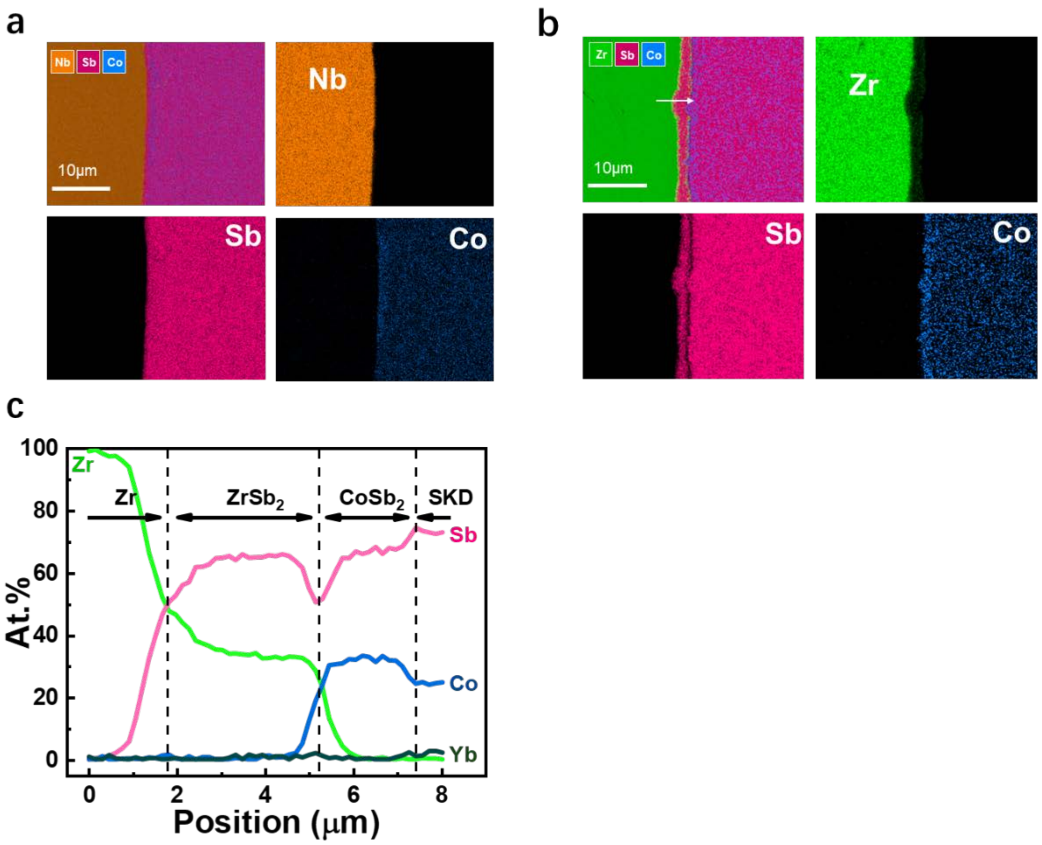

Supplementary Figure 3 Interfacial compositions for Nb joint and Zr joint. Elemental mapping images of as-prepared Nb joint (**a**), elemental mapping images (**b**) and compositional profile (**c**) (SKD: $Yb_{0.3}Co_4Sb_{12}$) at interfaces of as-prepared Zr joint.

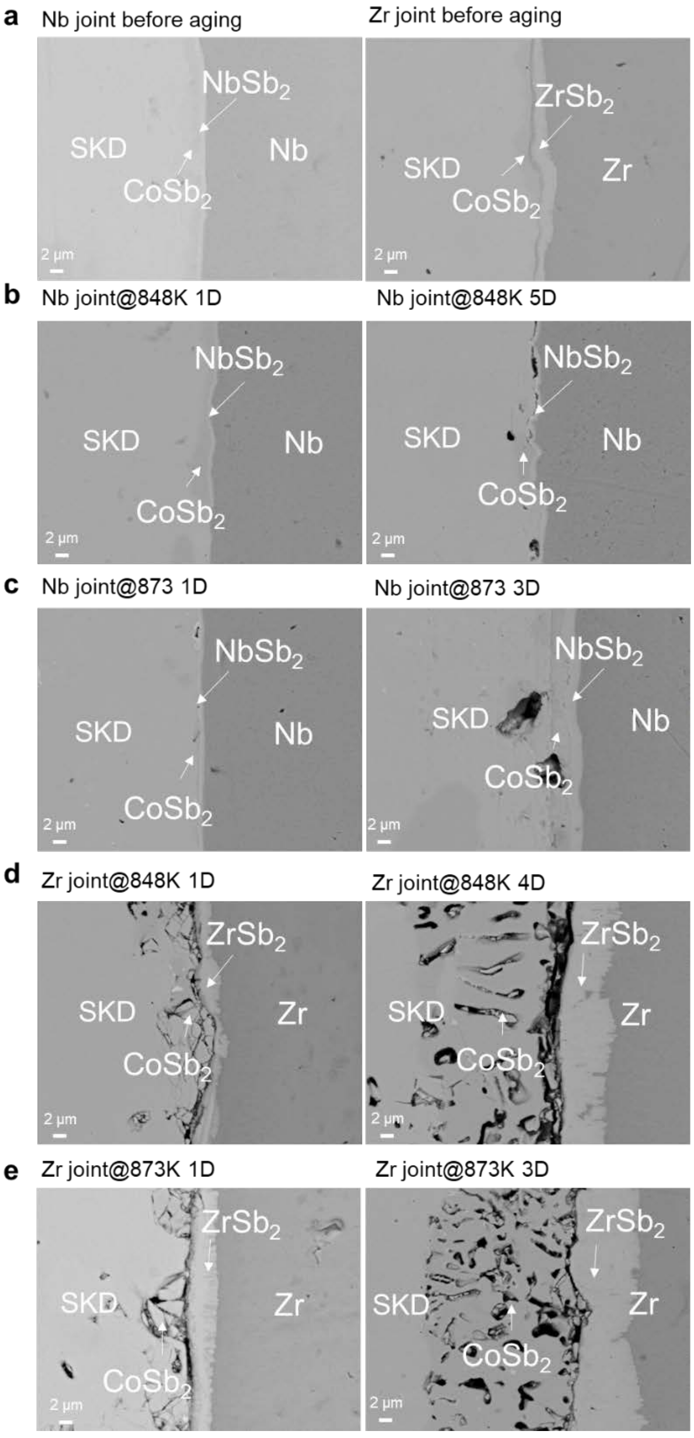

Supplementary Figure 4 Microstructures of interfacial evolution. **a** Nb joint and Zr joint before aging; **b** Nb joint aging at 848 K for 1 day and 5 days; **c** Nb joint aging at 873 K for 1 day and 3 days; **d** Zr joint aging at 848 K for 1 day and 4 days; **e** Zr joint aging 873 K for 1day and 3 days.

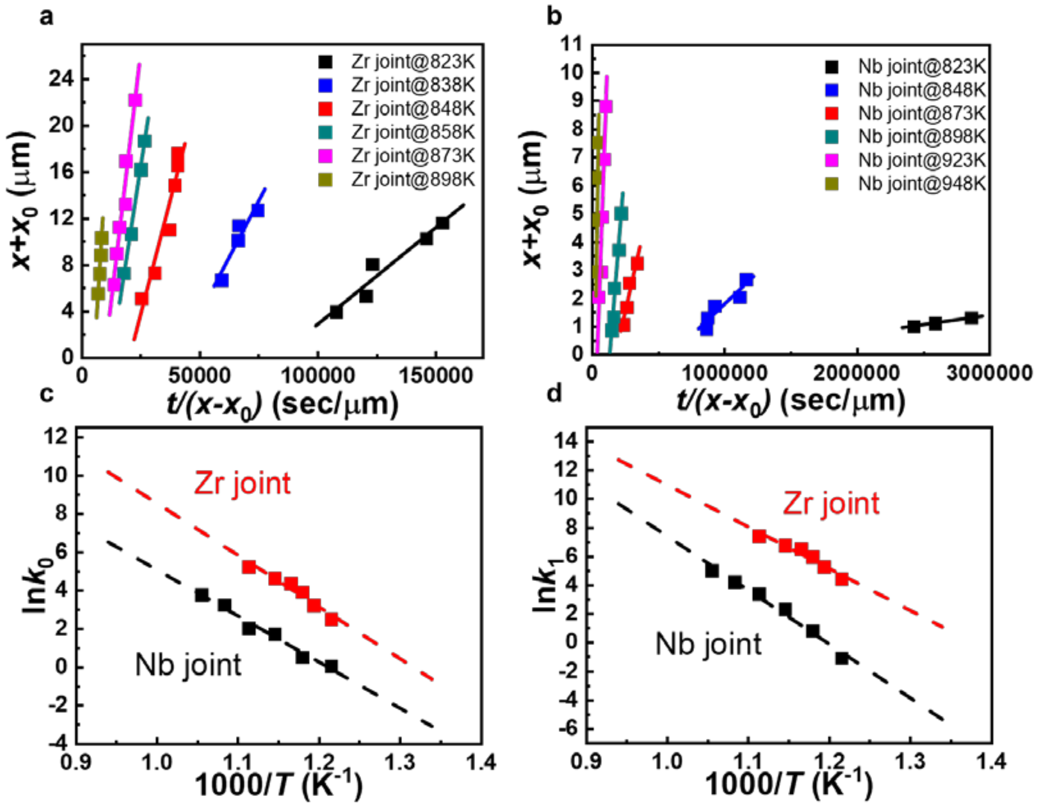

Supplementary Figure 5 Kinetic fitting details. Chemical constant (k_0) and diffusional constant (k_1) for Zr joint (**a**) and Nb joint (**b**). The straight line corresponds to the relation of $x + x_0 = 2k_1(\frac{t}{x-x_0} - \frac{1}{k_0})$, which is explained in Supplementary Note 1.The diffusional constant, k_1, can be found from the slope and the chemical constant, k_0, can be found from the y-axis intercept. The activation energy of chemical constant (E_0) **(c)** for Zr joint and Nb joint and activation energy of diffusional constant (E_1) **(d)** for Zr joint and Nb joint. The straight lines correspond to Arrhenius relation given as $lnk_0 = \ln(k_0^0) - \frac{E_0}{RT}$ and $lnk_1 = \ln(k_1^0) - \frac{E_1}{RT}$. The pre-exponential factors, k_0^0 and k_1^0, can be found from the y-axis intercept, and activation energies, E_0 and E_1, can be found from the slope. Error bars represent the standard deviations.

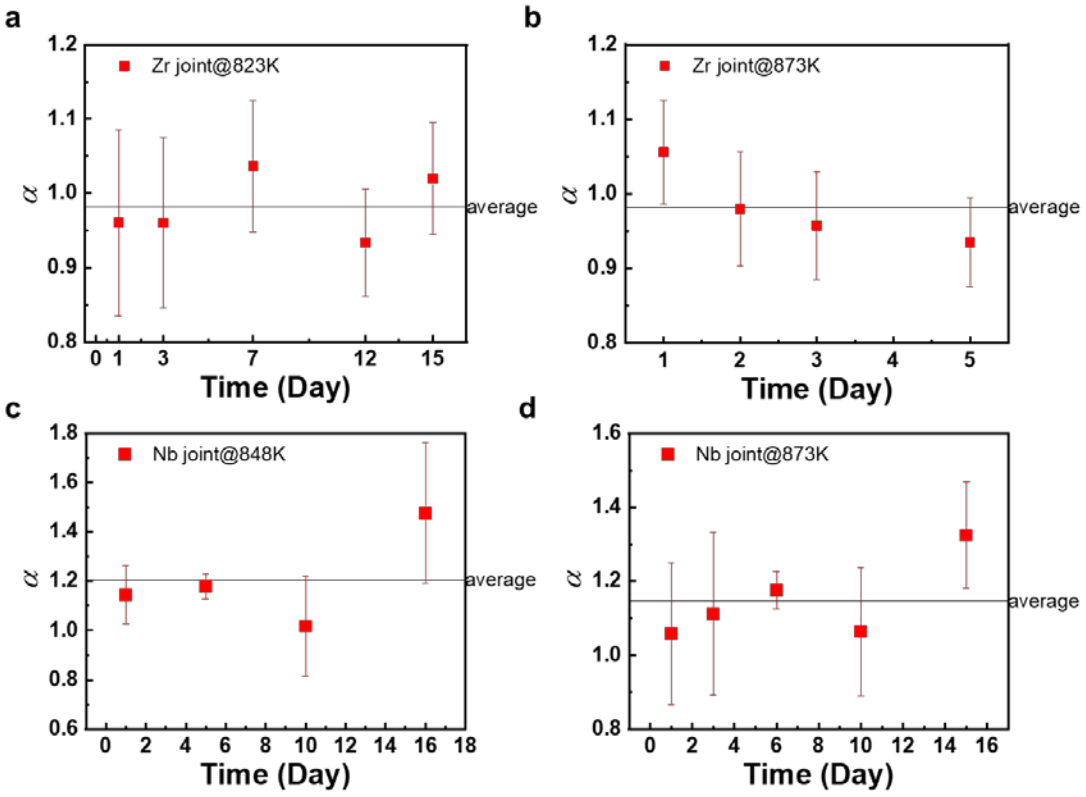

Supplementary Figure 6 The ratio (α) of the thickness of the decomposition layer, DL, (l_{DL}) to the thickness of the DL ($x\beta$) derived from the chemical reaction between $CoSb_3$ and barrier layer (Zr or Nb), $2CoSb_3 + Nb\ (Zr) = 2CoSb_2 + NbSb_2\ (ZrSb_2)$. The ratio ($\alpha$) for Zr joint aging at 823 K (**a**) and 873 K (**b**) and Nb joint aging at 848 K (**c**) and 873 K (**d**). α is around 1 in all aging temperatures and TE joints, indicating l_{DL} is approximately equal to $x\beta$ and further linearly related to x. Error bars represent the standard deviations.

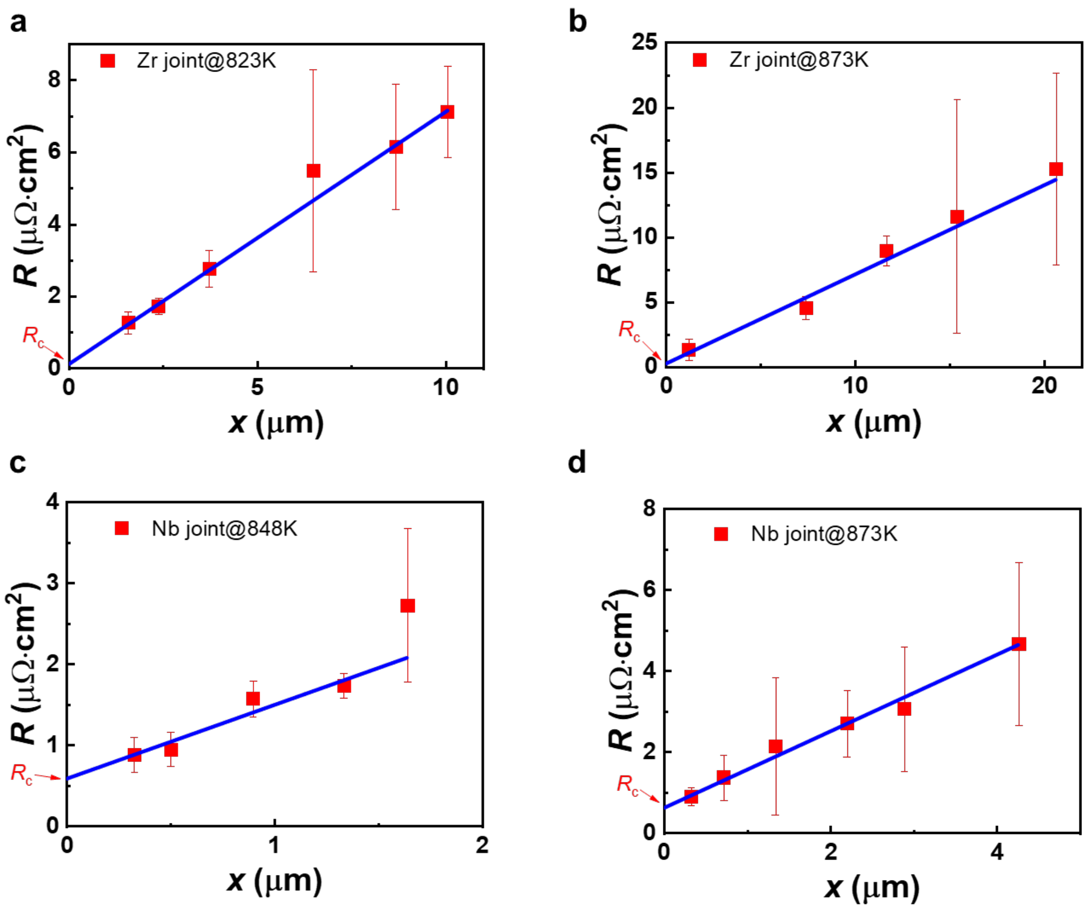

Supplementary Figure 7 The interfacial resistivity (*R*) as a function of the IRL thickness (*x*). The correlation for Zr joint aging at 823 K (**a**) and 873 K (**b**) and Nb joint aging at 848 K (**c**) and 873 K (**d**). The y-intercept of each fitting line indicates R_C. Error bars represent the standard deviations.

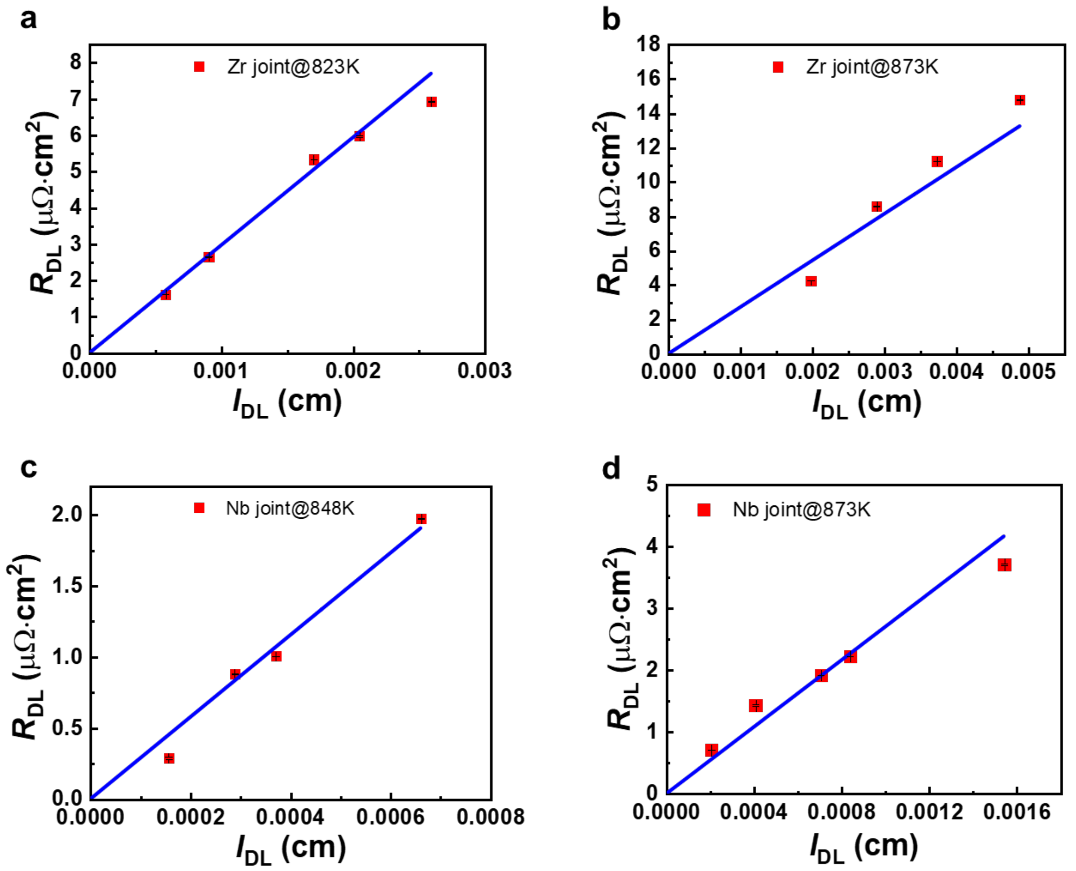

Supplementary Figure 8 The interfacial resistivity resulting from the decomposition layer (DL), R_{DL}, as a function of the thickness of the DL (l_{DL}). The correlation for Zr joint aging at 823 K (**a**) and 873 K (**b**) and Nb joint aging at 848 K (**c**) and 873 K (**d**). The slope of each fitting line indicates the resistivity of the DL, ρ_{DL}. The fitting line shall cross the origin since R_{DL} relies on the existence of the DL. Error bars represent the standard deviations.

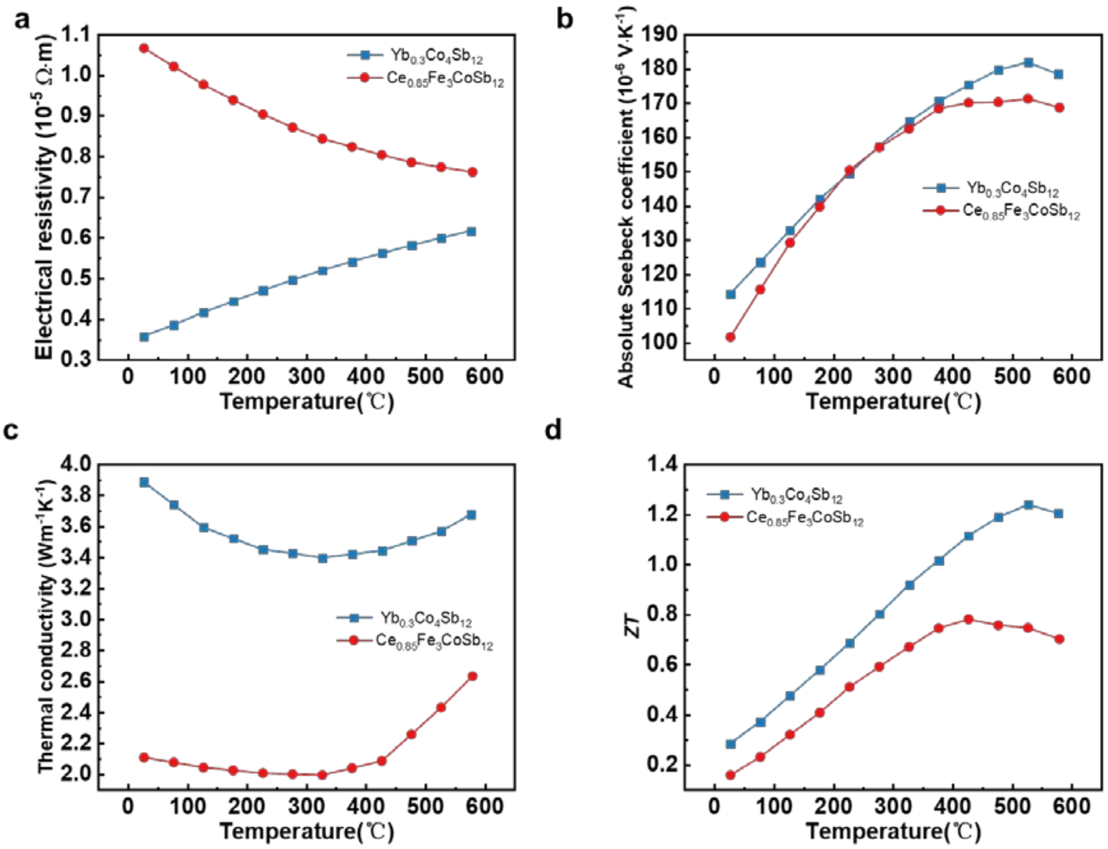

Supplementary Figure 9 The thermoelectric characterization for *n*-type SKD ($Yb_{0.3}Co_4Sb_{12}$) and *p*-type SKD ($Ce_{0.85}Fe_3CoSb_{12}$). Temperature dependence of the electrical resistivity (**a**), the absolute Seebeck coefficient (**b**), the thermal conductivity (**c**) and the dimensionless figure of merit ZT (**d**) for *n*-type SKD ($Yb_{0.3}Co_4Sb_{12}$) and *p*-type SKD ($Ce_{0.85}Fe_3CoSb_{12}$). The TE performances were characterized by commercial equipment (ZEM-3 and the Laser Flash method).

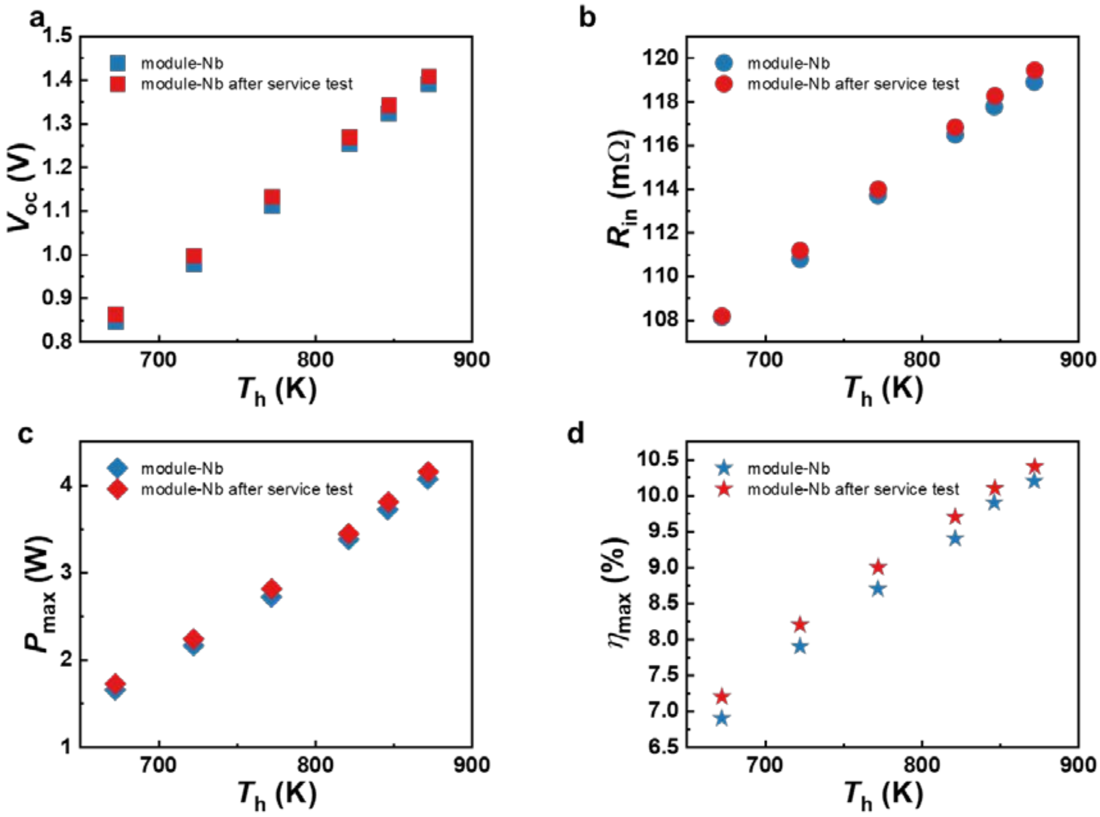

Supplementary Figure 10 Performance for the 8-pair module using Nb as the barrier layer before and after long-term service test. Open-circuit voltage (V_{oc}) (**a**), internal resistance (R_{in}) (**b**), maximum output power (P_{max}) (**c**), maximum conversion efficiency (η_{max}) (**d**) as a function of the hot-side electrode temperature (T_h) of the 8-pair module before and after long-term service test. The cold-side electrode temperatures of measurement for the module before long-term service test is 297 K and it is 293 K after the test.

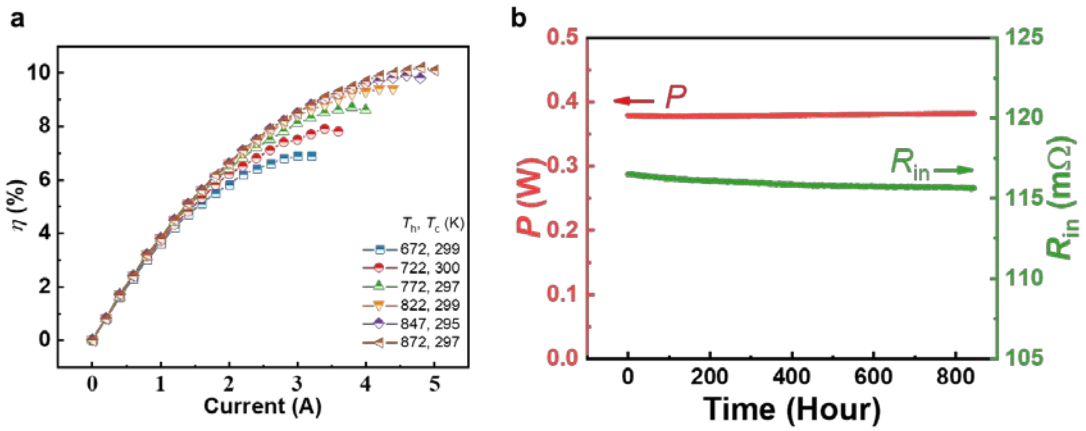

Supplementary Figure 11 Conversion efficiency and stability for the 8-pair module using Nb as the barrier layer. **a** Conversion efficiency (η) as a function of current at different operating temperatures of the 8-pair module using Nb as barrier layer. T_h and T_c represent the hot-side electrode temperature and the cold-side electrode temperature, respectively. **b** The time dependences of the output power (P) and the internal resistance (R_{in}) of the 8-pair module under the long-term service condition of hot-side temperature at 818 K and cold-side temperature at 308 K.

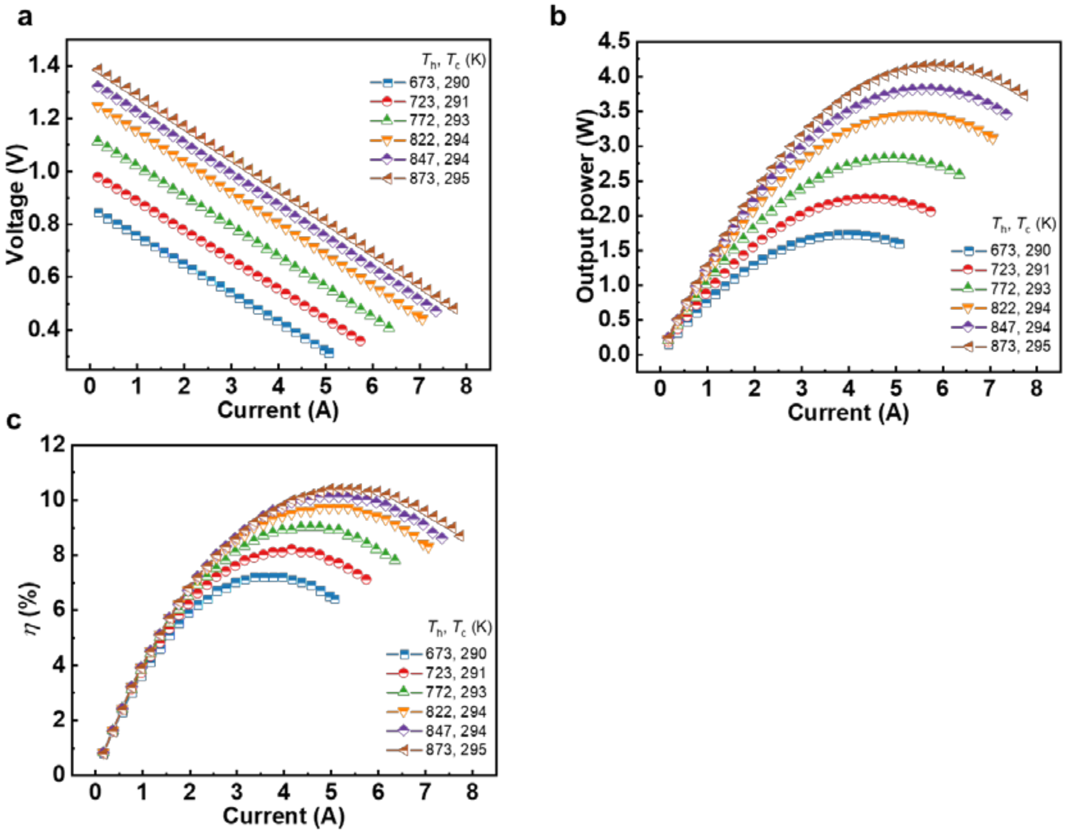

Supplementary Figure 12 Detailed data of the performance for the 8-pair module using Nb as the barrier layer after the long-term service test. Voltage (**a**), output power (**b**), conversion efficiency (η) (**c**) as a function of current at different operating temperatures of the 8-pair module after the long-term service test. T_h and T_c represent the hot-side electrode temperature and the cold-side electrode temperature, respectively.

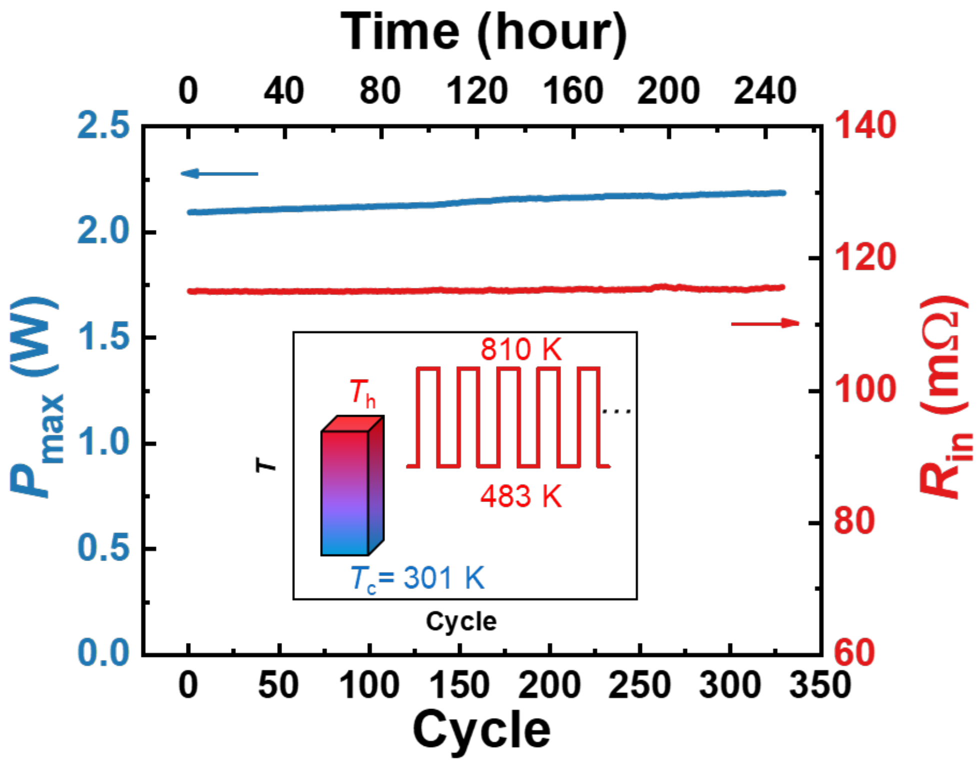

Supplementary Figure 13 The maximum output power (P_{max}) and the internal resistance (R_{in}) of the 8-pair module with Nb barrier layer under constant-thermal-shock condition that hot-side temperature (T_h) cycles between 810 K and 483 K and cold-side temperature (T_c) is fixed at 301 K. The P_{max} and R_{in} of the tested module show less than 4.2% and 0.5% changes, respectively, after 300 cycles.

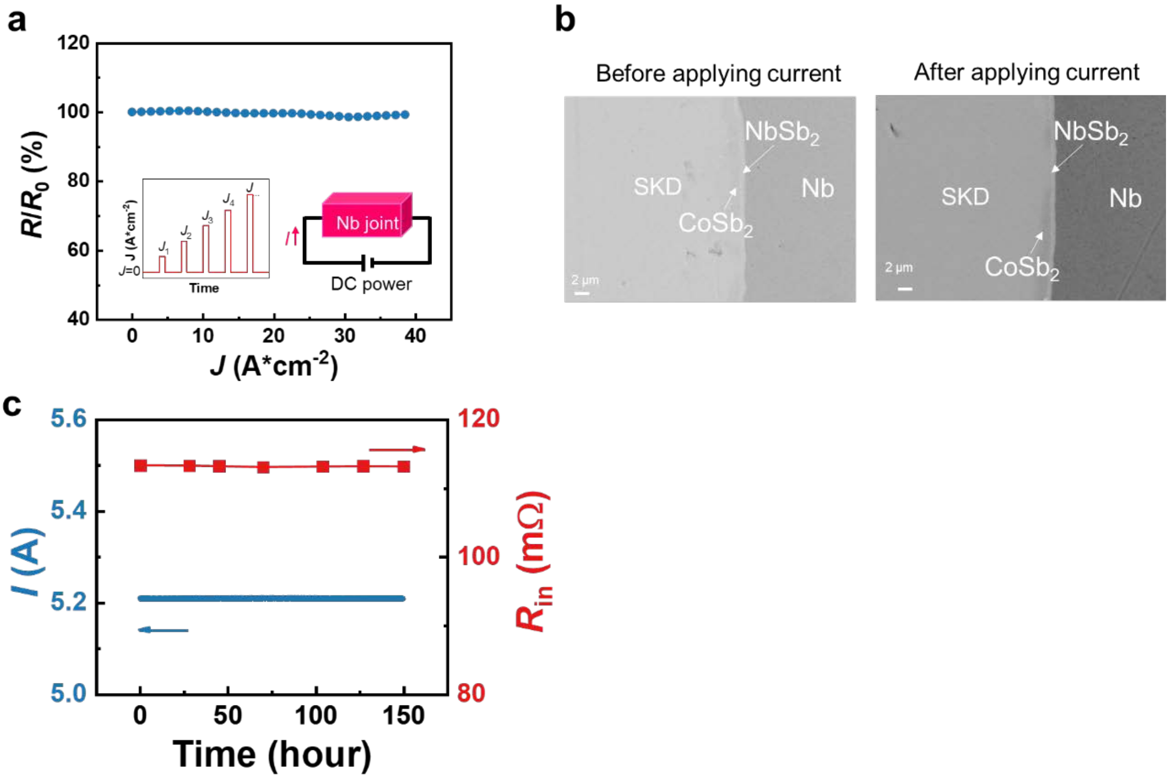

Supplementary Figure 14 Experimental results of the effect of current on the stability of Nb joint and the corresponding module. **a** Correlation between the current density (J) and relative electrical resistance variation (R/R_0) for Nb joint with length of 4 mm. **b** The interfacial microstructures for Nb joint before and after applying current test. **c** Time dependence of the internal resistance for SKD-based module with Nb barrier under the current of 5.2 A at the hot-side temperature of 773 K.

Supplementary Table 1 Stable compounds as products regarding to the interfacial reactions between $CoSb_3$ and *d*-metal layers, calculated interfacial reaction energy (E_{IR}) of corresponding interfacial reactions and activation energy barrier of Sb migration (E_{Mig}) through the interfacial product compounds.

Interfacial product compounds	E_{IR} (eV/atom)	E_{Mig} (eV/atom)
$NbSb_2$	-0.268	0.883
$TaSb_2$	-0.147	1.484
$ZrSb_2$	-0.56	0.556
Mo_3Sb_7	-0.012	0.806
$TiSb_2$	-0.393	0.736
$HfSb_2$	-0.417	0.610
YSb	-1.163	2.540
$AuSb_2$	-0.069	0.201
ScSb	-0.936	1.287

Supplementary Table 2 Values of R_C, R_{IRL}, ρ_{IRL}, R_{DL} and ρ_{DL} and the percentage of R_C, R_{IRL}, R_{DL} in total interfacial resistivity of Nb joint aging at 848 K and 873 K and Zr joint aging at 823 K and 873 K.

TE Joint	Aging temperature	Aging time	R_C		R_{IRL}		ρ_{IRL}	R_{DL}		ρ_{DL}
	(K)	(day)	(μΩ cm^2)	%	(μΩ cm^2)	%	(μΩ cm)	(μΩ cm^2)	%	(μΩ cm)
Nb	848	0	0.58	66	0.026	3	800[1]	0.28	31	2890
		1		61	0.04	4		0.3.	35	
		5		37	0.07	5		0.92	58	
		10		34	0.10	6		1.05	60	
		15		21	0.13	5		2.01	74	
	873	0	0.61	67	0.03	3		0.27	30	2710
		1		44	0.06	4		0.71	52	
		3		28	0.11	5		1.43	67	
		6		22	0.18	6		1.92	71	
		10		20	0.23	7		2.23	73	
		15		13	0.34	7		3.72	80	
Zr	823	0	0.10	8	0.02	1	120[2]	1.15	91	2970
		1		6	0.03	2		1.59	92	
		3		4	0.04	2		2.63	94	
		7		2	0.08	1		5.30	97	
		12		2	0.10	1		5.94	97	
		15		1	0.12	1		6.90	98	
	873	0	0.23	16	0.01	1		1.12	83	2720
		1		5	0.09	2		4.26	93	
		2		2	0.14	2		8.60	96	
		3		2	0.18	2		11.22	96	
		5		1	0.25	2		14.80	97	

Supplementary Note 1

Detailed Deal and Grove`s relation derivation

In the chemical reaction step, the time, $\mathrm{d}t_0$, of chemical transformations of the Sb atoms is directly proportional to the increase, $\mathrm{d}x$, of the thickness of the IRL and independent of its total thickness, x:

$$\mathrm{d}t_0 = \frac{1}{k_0}\mathrm{d}x, \quad (1)$$

where k_0 is the chemical constant ($\mathrm{m \cdot s^{-1}}$).

In the diffusion process step, the time, $\mathrm{d}t_1$, of diffusion of the Sb atoms is directly proportional to both the increase, $\mathrm{d}x$, of the thickness of the IRL and its existing total thickness, x:

$$\mathrm{d}t_1 = \frac{x}{k_1}\mathrm{d}x, \quad (2)$$

where k_1 is the physical (diffusional) constant ($\mathrm{m^2 \cdot s^{-1}}$).

The time, $\mathrm{d}t$, required for increasing the thickness of the IRL by $\mathrm{d}x$ is the sum of the time, $\mathrm{d}t_1$, of diffusion of the Sb atoms across the bulk to the reaction surface and the time, $\mathrm{d}t_0$, of their subsequent chemical interaction with the barrier atoms:

$$\mathrm{d}t = \mathrm{d}t_1 + \mathrm{d}t_0. \quad (3)$$

The subscript 1 at the diffusion time $\mathrm{d}t_1$ and the diffusional constant k_1 shows the diffusion process is related to x^1, while the subscript 0 at the chemical transformations time $\mathrm{d}t_0$ and the chemical constant k_0 indicates the chemical process is related to x^0.

Integration of Supplementary Equation 1 (initial condition $x = x_0$ at $t = 0$) gives

$$t = \frac{(x-x_0)^2}{2k_1} + \frac{x-x_0}{k_0}, \quad (4)$$

by which the IRL growth governed by both the chemical reaction and the atomic diffusion could be described.

Dividing both sides of Supplementary Equation 4 by $(x - x_0)$ gives

$$x + x_0 = 2k_1\left(\frac{t}{x-x_0} - \frac{1}{k_0}\right). \quad (5)$$

As x_0 can be taken with the plus sign, the experimental data should produce a

straight line in the coordinate $(x + x_0) - t/(x - x_0)$. In Supplementary Figure 5, the diffusional constant k_1 can be found from the slope of the straight line, while the chemical constant k_0 from its intercept on the ordinate axis.

Supplementary Note 2

The analysis of the interfacial resistivity

The interfacial resistivity can be divided into three parts, $R_{\mathrm{total}} = R_{\mathrm{IRL}} + R_{\mathrm{DL}} + R_{\mathrm{C}}$ (Fig. 4a). R_{DL} and R_{IRL} are the contribution from DL and IRL, respectively, and R_{C} represents the sum of contact resistivities contact of layers. According to the principle of interfacial resistivity measurement, R_{IRL} and R_{DL} can be calculated by layer thickness and electrical resistivity, $R_{\mathrm{IRL}} = \rho_{\mathrm{IRL}} \times x$ and $R_{\mathrm{DL}} = \rho_{\mathrm{DL}} \times l_{\mathrm{DL}}$, where x and l_{DL} are the thicknesses of IRL and DL, respectively. It is found that, the l_{DL} could be regarded as the thickness of TE material that decomposes when the corresponding IRL grows to a thickness of x (see Supplementary Fig. 6). Taking Nb joint as an example, according to the chemical reaction between the $CoSb_3$ and Nb, the molar ratio of $NbSb_2$ (IRL)to $CoSb_2$ (DL) is 1/2, which is equal to the value of

$$\frac{m_{\mathrm{NbSb_2}}/M_{\mathrm{NbSb_2}}}{m_{\mathrm{CoSb_2}}/M_{\mathrm{CoSb_2}}},$$

where m_{NbSb2} and M_{NbSb2} represent the mass and molar mass of $NbSb_2$, respectively, and m_{CoSb2} and M_{CoSb2} represent the mass and molar mass of $CoSb_2$, respectively. The mass can be represented by the product of the volume and density. Because the crossing area of all layers are the same, the volume can be replaced by the thickness. Therefore, the thickness of the DL can be given as:

$$2\frac{\rho_{\mathrm{NbSb_2}}}{M_{\mathrm{NbSb_2}}}\frac{M_{\mathrm{CoSb_2}}}{\rho_{\mathrm{CoSb_2}}}x = l_{DL}. \quad (6)$$

The volume difference in chemical reaction (β) is defined as

$$2\frac{\rho_{\mathrm{NbSb_2}}}{M_{\mathrm{NbSb_2}}}\frac{M_{\mathrm{CoSb_2}}}{\rho_{\mathrm{CoSb_2}}}$$

in Nb joint. In Zr joint, β would be

$$2\frac{\rho_{\mathrm{ZrSb_2}}}{M_{\mathrm{ZrSb_2}}}\frac{M_{\mathrm{CoSb_2}}}{\rho_{\mathrm{CoSb_2}}}.$$

The value of ρ_{IRL} can be equal to that of large bulk specimen because IRL grows without any visible defects[3]. However, because the DL contains obvious defects (see

Supplementary Fig. 4), the value of ρ_{DL} can only be determined from experimental data. Then the relation between R and x can be expressed as $R = (\rho_{\mathrm{IRL}} + \rho_{\mathrm{DL}} \times \beta) \times x + R_{\mathrm{C}}$ and its y-intercept is R_{C} (see Supplementary Fig. 7). Once R_{C} is obtained, R_{DL} and ρ_{DL} can also be determined (see Supplementary Fig. 8), since R and R_{IRL} have been determined.

Supplementary References

1. Schäfer H, Fuhr W. Beiträge zur chemie der elemente niob und tantal: XLIII. Verbindungen des niobs mit P, As, Sb, S, Se, Te. Synthese und chemischer transport. *Journal of the Less Common Metals* **8**, 375-387 (1965).

2. Garcia E, Corbett JD. Study of the crystal structures of ZrSb and β-$ZrSb_2$ and of the bonding in the two $ZrSb_2$ structures. *Journal of Solid State Chemistry* **73**, 452-467 (1988).

3. Tarento RJ, Blaise G. Studies of the first steps of thin film interdiffusion in the Al-Ni system. *Acta Metallurgica* **37**, 2305-2312 (1989).
